# Breast Surgeons’ Perspectives of Telehealth Visits for Breast Clinic

**DOI:** 10.1245/s10434-025-17936-z

**Published:** 2025-08-06

**Authors:** F. Dickerson, A. A. Wiener, C. Breuer, J. Schumacher, L. Bozzuto, L. Wilke, M. Lautner, H. Neuman

**Affiliations:** 1 Wisconsin Surgical Outcomes Research Program, University of Wisconsin, Madison, WI USA; 2 Department of Surgery, School of Medicine and Public Health, University of Wisconsin—Madison, Madison, WI USA; 3 Department of Surgery, University of North Carolina, Chapel Hill, NC USA; 4 University of Wisconsin Carbone Cancer Center, Madison, WI USA

## Abstract

**Background:**

Telemedicine has the potential to improve the quality of cancer care by improving access to care, but challenges with integrating telemedicine into surgical subspecialty practices exist. The objective of our study was to assess breast surgeons’ use and perspectives of telemedicine.

**Patients and Methods:**

Surgeon members of the American Society of Breast Surgeons anonymously completed a REDCap survey that elicited surgeons’ characteristics, telehealth experience over time, and perceived importance of physical exam. Descriptive statistics summarized surgeon characteristics. Chi-squared tests assessed the association between telehealth use and surgeon characteristics, and between physical exam importance and likelihood of future telehealth use.

**Results:**

We included 249 respondents; 82% of surgeons had experience with telehealth, and those who did not (*n* = 45, 18%) were more likely to be older (*p* < 0.001). Surgeons most commonly used telehealth for surgical planning (64%) and postop (61%) visits and least commonly used telehealth for new cancer (30%) and second opinion (25%) visits. Surgeons reported that the inability to perform a physical exam was a moderate/extreme problem for most visit types, and this was significantly associated with future telehealth use (*p* < 0.05 for all visit types).

**Conclusions:**

Although breast surgeons utilize telehealth, the inability to perform a physical exam was a significant limitation. Telehealth may have a role for visits where a physical exam is deemed less necessary. Future research should focus on patients’ telehealth perspectives and the impact of a physical exam on surgical planning in the virtual setting.

**Supplementary Information:**

The online version contains supplementary material available at 10.1245/s10434-025-17936-z.

Telemedicine has the potential to improve the quality of cancer care by improving access to care. In primary care and medical subspecialty settings, telemedicine has been reported to be comfortable, convenient, and efficient for both patients and providers.^1–3^ In addition, the use of telemedicine has been reported to expand access to care for many populations and to be comparable to in person visits for patients with barriers to care (e.g., those with low socioeconomic status and/or rural patients).^1,3^

Although there are many potential benefits to telemedicine, there are challenges to integrating telemedicine into surgical subspecialty practices. Surgery has a “hands on nature”^4,5^ and given the physical exam is central to many surgical visits for evaluating problems and symptoms, this is a notable barrier to using telemedicine. Even though some studies have found that surgical plans developed during telemedicine visits remained unchanged after in-person physical examination, more research is needed across surgical specialties, such as surgical oncology, where operative plans can change because of a patient’s clinical course, imaging, labs, symptoms, and physical exam.^6,7^ Finally, surgery requires a high level of trust between patient and surgeon, and there are concerns that the rapport needed may be difficult to foster through telemedicine.^1,7,8^ Consequently, uptake of telemedicine amongst surgical specialties has been slow compared with medical specialities,^4,5^ and focused mainly on the provision of postoperative care.^9,10^

The coronavirus disease-19 (COVID-19) pandemic provided a new opportunity for surgical subspecialties to explore the use of telemedicine. During the COVID-19 pandemic, synchronous audio/visual telemedicine visits^5^ were implemented to allow safe delivery of care to patients.^2,11^ This global event sped up the integration of telemedicine into surgical practice.

Given the potential benefits and challenges of integrating telemedicine into clinical practice, we were interested in understanding the experiences of breast surgeons with telemedicine in breast clinical practices. Breast care has unique aspects to it that may make telemedicine challenging, such as the need to establish rapport given the vulnerable and intimate nature of breast treatment decisions, along with the value of the physical exam in determining candidacy for surgery options. Surgical options for breast cancer include lumpectomy (also referred to as breast conservation therapy), various mastectomies (simple, modified radical, nipple-sparing, skin-sparing, and prophylactic), and breast reconstruction (using implants or the patient’s own tissue).^12–15^ Surgical choice is influenced by several factors, including breast size, tumor size and location, the presence of ptosis, and the patient's preferences and overall health.^12–15^ In this study, we surveyed breast surgeons who were members of the American Society of Breast Surgeons (ASBrS). The objective was to assess breast surgeons’ use and perspectives of synchronous audio/visual telemedicine.

## Patients and Methods

### Study Setting and Population

From 25 May 2023 to 14 July 2023, ASBrS members were sent an invitation to complete an anonymous online survey in REDCap. All respondents of the survey were surgeons who care for patients with breast cancer.

### Survey Characteristics and Dissemination

The full survey is available in the appendix. The survey included six questions that elicited surgeon characteristics and experience with telemedicine. Surgeon characteristics included age, years in practice, and fellowship training experience. We also assessed the characteristics of surgeons’ practices. Our survey inquired about respondent’s confidence using computer technology, and use of telemedicine in one’s clinical practice from March 2020 to present. For surgeons with telemedicine experience, additional questions elicited the visit types for which telemedicine was used during the COVID-19 pandemic (at the time of their highest telemedicine use), the percentage of total visits within each visit category that were telemedicine visits, and whether they would use telemedicine for each visit type in the future. The nine visit types included new patient: benign, new patient: cancer, new patient: high risk, second opinion, surgical planning, postoperative, long-term follow-up, multidisciplinary, and others. We also asked questions about how telemedicine was integrated into clinical workflow and the extent to which the inability to perform a physical exam for each visit type was perceived to present a problem. We asked surgeons to rate on a scale from 0 to 10 (0 being negative and 10 being positive) the perceived impact of telemedicine on a variety of factors relevant to breast clinical practice (for example, use of clinic space, capacity to involve support people into visit, and establishment of rapport). The survey ended with two open-ended questions about innovative use of telemedicine and telemedicine pitfalls.

The survey was pilot tested with the University of Wisconsin, Department of Surgery, Breast Surgery Group and iteratively revised prior to distribution.

### Statistical Analysis

We used descriptive statistics to summarize surgeon characteristics. Chi-squared tests were used to assess the association between surgeon characteristics and use of telemedicine, including telemedicine use during the COVID-19 pandemic, current use, and future use for each of the nine distinct visit types. Breast surgeons’ perceptions of the effect of telemedicine on various clinical factors were summarized with median and interquartile ranges. Chi-squared tests were also used to assess the relationship between the perceived importance of physical exams and the likelihood of future telemedicine use for each of the visit types. All analyses were performed using Stata v18.

## Results

### Demographics

Of the 267 surgeons that opened the survey, 17 did not answer any questions and 1 only answered surgeon characteristic questions (final sample size *n* = 249). Survey respondents varied in age (Table 1). Most surgeons had been in practice for 20+ years (52%, *n* = 130). In addition, most surgeons reported that breast surgery made up the majority of their practice (82% stated that greater than 80% of their practice is dedicated to breast), and the majority had fellowship training (55%) (Table 1). Respondents represented varied practice types, regions of the country, and rural/urban practice locations (Table 1).

**Table 1:** Characteristics of respondents (N=249)

Characteristics of respondents	N (%)
*Age*	
< 45	63 (25%)
46–55	67 (27%)
56–65	75 (30%)
> 65	43 (17%)
*Years in practice*	
0–10	45 (18%)
10–20	71 (29%)
> 20	130 (52%)
*Fellowship training*	
Breast	111 (45%)
Surgical oncology	24 (10%)
Other	15 (6%)
None	97 (39%)
*Practice environment*	
Academic	71 (29%)
Hospital owned (academic affiliated)	53 (21%)
Hospital owned (no affiliation)	56 (22%)
Private practice	60 (24%)
Other	8 (3%)
*Region of the country*	
West	48 (19%)
Midwest	51 (20%)
Northeast	75 (30%)
South	70 (28%)
Non-US	2 (1%)
*County population of main practice*	
Large urban center (> 1 million)	88 (36%)
Medium-large urban area (250,000–1 million)	87 (35%)
Medium urban area (20,000–250,000)	57 (23%)
Small urban area or rural (< 20,000)	16 (6%)

Overall, most (82%, *n* = 204) breast surgeons reported using telemedicine in their practice. Further, 88% (*n* = 220) of breast surgeons stated that they were comfortable and confident when using computer technology.

### Perspectives of Surgeons with No Telemedicine Experience

A total of 45 respondents (18%) stated they had no personal experience with telemedicine. Surgeons who did not have experience with telemedicine were more likely to be older (*p* < 0.05), in practice for 20 or more years (*p* < 0.05), and in private (rather than academic-affiliated) practice (*p* < 0.05). The most common factor reported for not using telemedicine was the inability to perform a physical exam. Many of these respondents were open to using telemedicine in the future, especially for multidisciplinary (81%) and surgical planning visits (77%).

### Perspectives of Surgeons with Experience Using Telemedicine in Clinical Practice

#### Visit Types and Telemedicine Use during COVID-19

**Fig. 1:**
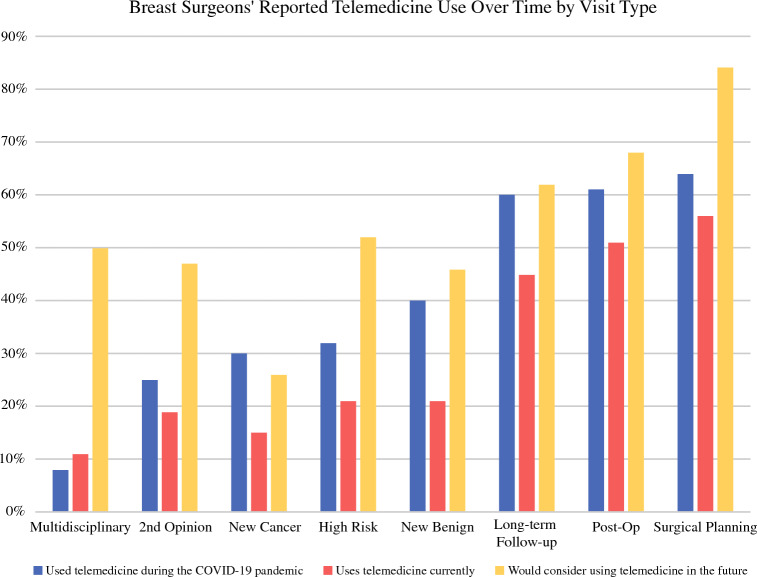
Surgeons’ report of telemedicine use during the COVID-19 pandemic, in current clinical practice, and potential future use for the most common visit types (“other” is not included as a separate visit type as the visits listed fell into the other prespecified categories or were not applicable)

For surgeons with experience using telemedicine in clinical practice (*n* = 204; 82%), there was variation in the visit types for which it was utilized. During the pandemic, telemedicine was most commonly used for surgical planning follow-up visits (64%), postoperative visits (61%), and long-term follow-up visits (60%). Telemedicine was less commonly used for new patient cancer visits (30%), second opinion visits (25%), and multidisciplinary visits (8%). Figure 1 summarizes surgeon reported prior (during the pandemic), current (survey conducted in May–July 2023), and potential future use of telemedicine for each visit type. For most visit types, respondents’ use of telemedicine during the COVID-19 pandemic was greater than their current use. In addition, breast surgeons indicated that, compared with current use for each visit type, they would be more likely to use telemedicine in the future. Respondents reported that they were most likely to consider using telemedicine in the future for surgical planning visits (84%), postop visits (68%), and long-term follow-up visits (62%). Surgeons with experience using telemedicine for a particular visit type during the pandemic were more likely to report current and probable future use for that visit type (*p* < 0.05 for each visit type).

#### Perceived Importance of Physical Exam in Context of Telemedicine Use

**Fig. 2:**
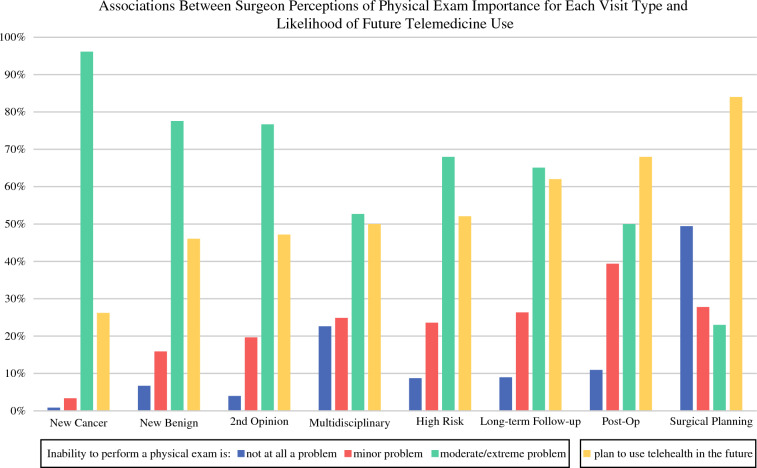
Associations between surgeon perceptions of physical exam importance for each visit type and likelihood of future telemedicine use

Most surgeons reported that the inability to perform a physical exam was a moderate/extreme problem for most visit types (Fig. 2). The inability to do a physical exam was perceived to be the greatest problem for new patient cancer visits (96%), new patient benign visits (78%), second opinion visits (76%), high-risk visits (68%), and long-term follow-up visits (65%) (Fig. 2). The perceived importance of a physical exam was significantly associated with the likelihood of future use of telemedicine (Fig. 2, *p* < 0.05 for all visit types).

#### Perceived Impact of Telemedicine Use on Clinical Practice

**Fig. 3:**
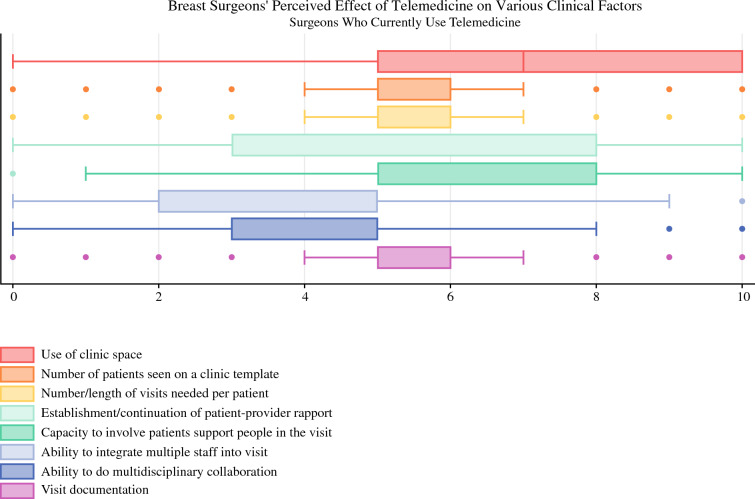
Breast surgeons’ perceived effect of telemedicine on various clinical factors; each factor was rated on a scale from 0 to 10, with 10 being the most positive and 0 being the most negative; each box spans the interquartile range representing the middle 50% of the data with the left edge of the box corresponding to the first quartile and the right the third quartile; the lines represent the range of responses

Most surgeons incorporated telehealth visits interspersed throughout their clinic day (54%, *n* = 110), rather than clustered or on a separate clinic day. Surgeons varied in their perspectives of the length of time of a telemedicine visit compared with an in-person visit, with 34% reporting it took the same amount of time, 16% reported it took more, and 49% reporting it took less. We assessed surgeons’ perspectives on the impact of telemedicine use on clinical practice (Fig. 3). Surgeons were relatively neutral about the impact of telemedicine on their clinical practice. Surgeons perceived that telemedicine could have a positive impact on clinic space (median 7, IQR 5, range 0–10). In addition, some surgeons perceived the ability to incorporate support people into the visit as a positive (median 7, IQR 3, range 0–10). Surgeons had varied perceptions of how telemedicine could impact establishment of rapport with patients (median 5, IQR 5, range 0–10).

### Open-Ended Response Questions Regarding Benefits and Challenges of Telemedicine

We asked current telemedicine users open-ended response questions regarding the perceived benefits and pitfalls of telemedicine (Table 2). Several surgeons described their experience providing postoperative care via telemedicine as positive and patient-centered, sparing patients the cost and time associated with travel, and increasing flexibility in scheduling. In addition, surgeons commented that telemedicine allowed them to gain valuable insight into patients’ home lives and easily incorporate patients’ support systems into the visit. Finally, surgeons viewed the capacity to bill for care that was previously performed via telephone as a benefit.

**Table 2:** Potential benefits and challenges of using telemedicine with breast surgery clinics

Themes	Surgeon comments
Post-op visits well-suited to telemedicine and patient-centered	I do almost all my post ops and post core visits via video visit. Patients love that they don’t have to drive to the office and can do the 10–15 min follow up without taking off from work. I discuss that I will offer a video visit with them pre op or pre bx to prepare them for it. I let them know that I will ask to see the wound on camera, and they should have a private space to talk. They always have the option of an in person visit, but > 90% select virtual Not sure it is innovative but I currently schedule all post-ops as telemedicine (unless they need a drain pull). A good number of my patients travel from a distance and this has facilitated those visits and reduced their costs associated with travel for in person visits We had a couple in their 80s demonstrate enthusiasm for video consultation with med one because it saved them a drive/reduced their exposure
Allows insight into home situation and integration of caregivers into consultation	The ability to see the patient in their home—when they turn on the camera—adds to the information about the patient that cannot be gained during an office appointment To bring in other relatives who are in other states the get involved in the discussions I use telemedicine to include family members of elderly pts and to review imaging findings that requires biopsy or additional work up
Enhanced opportunity to bill for activities that previously done via telephone	I previously reviewed staging MRI breast with patient by phone to finalize surgical plans during that call, and reminded pt about details of surgery. Now I do that call as a billed Telemed visit with is more convenient to me and patient since I don't need to repeat physical exam
Challenging to ensure privacy	Patient distracted by other things at home and not hearing the treatment plan It is hard to ensure the patient has privacy
Sensitive nature of the breast practice	Having the patient disrobe on camera is uncomfortable for them and inadequate to assess a problem So often in breast surgery, physical examination is key. 'Hold the phone a little closer to your breast' just doesn’t cut it. While I have used telemedicine in my general surgery practice, predominantly by phone only and for routine post-op visits, I haven’t been able to see an easy way to use it in my breast practice
Scheduling obstacles	Having interspersed telemedicine appts during an in person clinic is a huge time waster. The staff have a hard time engaging when I switch back and forth, and there is no one available for patient tech support…. I plan to do clustered visits for improved scheduling in clinic

Surgeons also described some challenges with telemedicine (Table 2). One surgeon described that patients are distracted from the visit when they are at home. Additional challenges included patients not having a private place to participate in the visit, and patients feeling uncomfortable undressing on camera (particularly relevant for breast clinics). Other providers mentioned that visits are often scheduled inefficiently within current clinic templates, highlighting that clustered visits are preferred to interspersed visits. Another major pitfall highlighted was difficulty with technology, which surgeons noted could manifest in various forms—some patients do not have internet access (e.g., rural, elderly), some patients do not own a computer/tablet, and some patients are not fluent in English and online translation during telemedicine appointments can be challenging. Lastly, if a complication is discovered during a postoperative telemedicine visit, it cannot be treated right away; thus, an additional in-person visit would have to be scheduled, causing a delay in care (e.g., seroma aspirations), and creating additional patient costs.

## Discussion

In this survey study of breast surgeon ASBrS members, we found that most breast surgeons reported that they use telemedicine in their practice and are comfortable and confident using it. Surgeons reported using telemedicine for a variety of visit types. Visits where the physical exam was perceived to be less important, such as surgical planning visits, postoperative visits, and long-term follow-up visits, were viewed as the most appropriate for telemedicine. Although surgeons’ current use of telemedicine was reported as lower than during the peak of the COVID-19 pandemic, most respondents remained open to using telemedicine within their practice in the future for an expanded range of visit types. This willingness, paired with advances in clinical technology, indicates that telemedicine will be an important tool for enhancing efficiency and access to clinic visits moving forward.

Telemedicine has the potential to improve access for patients, especially those who have challenges physically making it to clinic. Contributing challenges could include the distance they reside from the clinic, weather impacting travel, access to transportation/parking, their work schedule, and their current health status. Telemedicine could be a way to increase and improve access to follow-up care in rural or underserved communities,^16–18^ and some studies have even found a decreased rate of patient no-shows when telemedicine appointments are offered.^10^ Telemedicine may also provide an avenue where other clinical team members, and patients’ support systems, could be included.

Our findings in this study are consistent with prior work examining physicians’ perspectives regarding telemedicine.^2,19^ These studies have noted an increased use of telemedicine,^2^ especially for physicians who are more adaptable, are confident with technology use/troubleshooting, and who primarily practice in an outpatient setting.^2^ In these studies, surgeons have generally been less likely to use telemedicine.^2^

However, our study supports that surgeons are open to incorporating telemedicine into their clinical practice, though use depends on the visit type.^11,20^ In a retrospective review of general surgery patients within Veterans’ Affairs (VA), telemedicine was observed to be effective and safe when used for common general surgery procedure postoperative visits.^21^ In addition, a 2023 systematic review of patients who underwent surgical oncology resections and had at least one virtual long-term follow-up visit found that telemedicine was favorable for patients and providers, and that cancer recurrence and readmission rates were similar among those who participated in telemedicine visits compared with in-person follow-up visits.^9^ Our survey study observations were consistent with this research in that surgeon respondents were most likely to use telemedicine for visits with established patients and for postoperative visits.^19^

A significant driver of breast surgeons’ comfort when using telemedicine in practice relates to the perceived importance of the physical exam. When a patient is new to a surgeon, the physical exam seems to be of the upmost importance, as the surgeon must establish a baseline to inform decision-making. This is consistent with the literature, with some studies noting that even oncology patients themselves have had concerns about the lack of physical examinations during telemedicine visits.^16,22^ Our study found that a physical exam was perceived to be less critical for surgical planning and postoperative appointments, likely because a baseline physical exam had already been performed, and the visit was focused on the conversation and decision-making. Telemedicine may have a role in breast cancer clinics for surgical planning visits, and potentially for postoperative visits depending on whether that postoperative visit focuses more on discussing pathological results and next steps versus requiring a physical exam.

A significant challenge limiting the integration of telemedicine into clinical practice is related to billing. Providers have acknowledged the potential efficiency and cost-saving benefits of telemedicine,^11,16,20,22^ and efficiency was a key theme in our free text responses. However, the ability to bill for care provided via telemedicine is inconsistent. One of our surgeon respondents listed concerns about billing in their free text response, stating that they “have not been able to successfully incorporate tele-visits owing to billing restrictions and issues with staff understanding.” Further, they mentioned that during the COVID-19 pandemic, telemedicine was able to be better utilized owing to lifting the restriction on patient location and physician licensure; therefore, all patients could be cared for remotely even if they “were located on a different coast and in different countries.” There was an authorized extension of “no geographic restrictions” for Medicare patients who receive telehealth for nonbehavioral or nonmental health visits through 31 March 2025, but this does not include all insurance types and longevity will depend on future legislation.^7,23^ Since some of the geographic restrictions have been reinstated, it is likely that this has contributed to the decrease in telemedicine use that we observed in our study. Permanently lifting patient location restrictions may be a solution that could help many communities access care. However, one caveat to consider is the need for reliable internet and access to technology that many of those groups may be lacking.^17^

A strength of our study was that we had a diverse pool of respondents who worked in different practice environments from different regions of the USA. There was enthusiasm from providers regarding telemedicine on the basis of the answers to the open-ended questions in particular. We designed our survey to focus largely on how telemedicine impacted clinical practice but did not include any questions eliciting perspectives about patient populations that might benefit most from telemedicine. However, the free text responses allowed us to gain insight into this topic, as providers had opinions on innovative ways to use telemedicine in various communities. This was especially true for rural and underserved communities, and for the elderly.

There are limitations to our work. First, our survey represented the perspectives of surgeons, and we did not talk to patients with breast disease. Second, although we captured the self-reported characteristics of providers’ practices, some of the questions did not provide sufficient granular detail to understand how practice setting influences telemedicine use. For example, we asked surgeons about the population of the county of their main practice, but this question does not capture the catchment area of the practice (e.g., the proportion that must travel from a more rural area for whom telemedicine may be beneficial). Finally, our study had the limitation of survey-response bias. Because our survey was distributed anonymously to the society membership, whose population is fluid owing to changes in membership status, we cannot calculate an exact response rate. It is possible that the perspectives in the study cannot be generalized to the broader population of breast surgeons. Despite these limitations, we believe that our survey provides valuable insight into how telemedicine can be incorporated into breast surgical practices.

## Conclusions

In this survey of breast surgeons within the ASBrS, we found that most surgeons utilized telemedicine during the COVID-19 pandemic and would be interested in using telemedicine in the future for visits where a physical exam is considered to be less important. Breast surgeons’ comfort and willingness to use telemedicine currently and in the future, show that it is a valuable tool that could be used for increasing clinic visit efficiency and accessibility. While some research has found that telemedicine visits allow equitable access to care across demographics and for those who are socially vulnerable,^16^ more research is needed on the patient perspective, specifically vulnerable populations such as rural populations, geriatric patients, and those of lower socioeconomic status. Further work could help determine how and whether telemedicine can improve access to these diverse populations.

## Supplementary Information

Below is the link to the electronic supplementary material.

Supplementary file1 (DOCX 38 kb)
