# Supplementary material for: Breast Surgeons’ Perspectives of Telehealth Visits for Breast Clinic
Source: Ann Surg Oncol. 2025 Aug 6;32(11):8463–71. doi: 10.1245/s10434-025-17936-z (PMC12494603; doi:10.1245/s10434-025-17936-z)
Supplement: Supplementary file 1 — Supplementary file1 (DOCX 38 kb) [file 10434_2025_17936_MOESM1_ESM.docx]

# Synchronous Audio/Video Visit Telemedicine Use in Breast Surgery Survey

# Surgeon demographics

1. What is your age?

- <35
- 35-45
- 46-55
- 56-65
- >65
- Prefer not to answer

1. How many years have you been in practice?

- Still a resident trainee
- Still in fellowship training
- 0-10
- 10-20
- >20
- Retired

1. What is your fellowship training experience?

- Breast fellowship
- Surgical oncology fellowship
- Fellowship other than breast or surgical oncology
- No fellowship

# Practice details

1. What proportion of your practice is dedicated to breast surgery?

- <20%
- 20-40%
- 41-60%
- 61-80%
- >80%

1. If you mostly practice in the US, in what region do you practice?

- West
- Midwest
- Northeast
- South
- Non-US practice

1. What is the population in the county of your main practice?

- Large urban center (>1 million)
- Medium-large urban area (250,000-1 million)
- Medium urban area (20,000-250,000)
- Small urban area or rural (<20,000)

1. Which of the following best describes your main practice environment?

- Academic medical center
- Hospital owned practice (academic-affiliated)
- Hospital owned practice (not academic-affiliated)
- Private practice
- Other

# Telemedicine Usage

1. Indicate your agreement/disagreement with the following statement:

I feel comfortable and confident when using computer technology.

- Strongly Agree
- Agree
- Neutral
- Disagree
- Strongly Disagree

1. From March 2020 to the present, have you used telemedicine in your clinical practice? For this survey, we define telemedicine as *synchronous video and/or audio clinic visits with patients.*

- Yes
- No

1. What other providers in your clinical practice utilize telemedicine? Select all that apply

- Breast Cancer Nurse Navigators
- Advanced practice providers (Physician Assistants / Nurse Practitioners)
- Social workers
- None

**If no to question 9, skip to page 8.**

**If yes continue:**

1. For which visit types did you use telemedicine during the COVID19 pandemic at the time of your highest telemedicine use? Select all that apply
   - New patient: benign
   - New patient: malignant
   - New patient: high risk
   - New patient: second opinion
   - New patient: follow-up decision making discussion
   - Post-op
   - Long-term follow-up
   - Multi-Disciplinary (joint visit with Med onc/Rad onc)
   - Other (free text)

# Perceptions of telemedicine

1. We are interested in understanding breast surgeons’ perspectives on the impact of telemedicine on breast surgical practice, including both positive and negative effects.

Please rate the *effect of telemedicine* on the following factors on a scale of 0-10 (0= very negative effect, 5= neutral, 10= very positive effect)

|  | **Very negative effect** |  |  |  |  | **Neutral** |  |  |  |  | **Very positive effect** |
| --- | --- | --- | --- | --- | --- | --- | --- | --- | --- | --- | --- |
|  | **0** | **1** | **2** | **3** | **4** | **5** | **6** | **7** | **8** | **9** | **10** |
| Use of clinic space |  |  |  |  |  |  |  |  |  |  |  |
| Number of patients seen on a clinic template |  |  |  |  |  |  |  |  |  |  |  |
| Number/length of visits needed per patient |  |  |  |  |  |  |  |  |  |  |  |
| Ability for patients to participate in clinic visits |  |  |  |  |  |  |  |  |  |  |  |
| Establishment/continuation of patient-provider rapport |  |  |  |  |  |  |  |  |  |  |  |
| Capacity to involve patient’s support people in the visit |  |  |  |  |  |  |  |  |  |  |  |
| Ability to integrate multiple staff into visit (eg. medical assistant, nurse, interpreters) |  |  |  |  |  |  |  |  |  |  |  |
| Ability to do multidisciplinary collaboration |  |  |  |  |  |  |  |  |  |  |  |
| Visit documentation |  |  |  |  |  |  |  |  |  |  |  |
| Other (free text) |  |  |  |  |  |  |  |  |  |  |  |

1. Please indicate the percentage of your patient visits in each category that are **currently** conducted via telemedicine and whether you would consider using telemedicine for the visit type in the **future** (post-pandemic or in endemic phase).

|  |  | **Percent of total clinic visits of each visit type that are currently telemedicine** | | | | **I don’t see this visit type** | **Would you use telemedicine for the visit type in the future?** | |
| --- | --- | --- | --- | --- | --- | --- | --- | --- |
|  |  | **0%** | **<10%** | **10-50%** | **>50%** |  | **Yes** | **No** |
| **Visit type** | New patient: benign |  |  |  |  |  |  |  |
|  | New patient: malignant |  |  |  |  |  |  |  |
|  | New patient: high risk |  |  |  |  |  |  |  |
|  | New patient: second opinion |  |  |  |  |  |  |  |
|  | New patient: follow-up, decision making discussion |  |  |  |  |  |  |  |
|  | Post-op |  |  |  |  |  |  |  |
|  | Long-term follow-up |  |  |  |  |  |  |  |
|  | Multi-Disciplinary (joint visit with Med onc/Rad onc) |  |  |  |  |  |  |  |
|  | Other |  |  |  |  |  |  |  |

1. How do you incorporate telemedicine visits into your clinic schedule currently? Select all that apply

- Intersperse with in-person template
- Clustered virtual visits at certain points in the day
- Separate virtual-only clinic
- Other (free text)

1. For a given visit type, the average time it currently takes you to do a telemedicine versus an in-person visit is:

- The same amount of time
- More time to do telemedicine
- Less time to do telemedicine

1. Based on your experience, to what extent is inability to perform a physical exam a problem for the following visit types?

|  |  | **Not at all a problem** | **Minor problem** | **Moderate problem** | **Extreme problem** | **I don’t see this visit type** |
| --- | --- | --- | --- | --- | --- | --- |
| **Visit type** | New patient: benign |  |  |  |  |  |
|  | New patient: malignant |  |  |  |  |  |
|  | New patient: high risk |  |  |  |  |  |
|  | New patient: second opinion |  |  |  |  |  |
|  | New patient: follow-up, decision making discussion |  |  |  |  |  |
|  | Post-op |  |  |  |  |  |
|  | Long-term follow-up |  |  |  |  |  |
|  | Multi-Disciplinary (joint visit with Med onc/Rad onc) |  |  |  |  |  |
|  | Other |  |  |  |  |  |

# Open response

1. Drawing on the collective wealth of experience during the COVID pandemic, we are interested in compiling innovative uses of telemedicine that may improve care quality. Please describe an innovative use of telemedicine that you have used in your practice. Please include any relevant details that may help another provider replicate this use in their practice.
2. We are also interested in informing the community about potential telemedicine pitfalls, so breast surgeons can fully consider their telemedicine strategy. Please describe an unsuccessful use of telemedicine that you believe should not be tried again. Please include any relevant details about the specific use and why you think it was unsuccessful.

# Telemedicine non-use

1. What was the single most important factor that contributed to your non-use of telemedicine?
2. Would you be open to using telemedicine in the future?

- Yes
- No
- Not sure

**If no, skip to page 9.**

**If yes or not sure continue:**

1. Would you envision implementing telemedicine for the following visit types?

|  |  | **Definitely yes** | **Probably yes** | **Probably not** | **Definitely not** |
| --- | --- | --- | --- | --- | --- |
| **Visit Type** | New patient: benign |  |  |  |  |
|  | New patient: malignant |  |  |  |  |
|  | New patient: high risk |  |  |  |  |
|  | New patient: second opinion |  |  |  |  |
|  | New patient: follow-up, decision making discussion |  |  |  |  |
|  | Post-op |  |  |  |  |
|  | Long-term follow-up |  |  |  |  |
|  | Multi-Disciplinary (joint visit with Med onc/Rad onc) |  |  |  |  |
|  | Other |  |  |  |  |

# Perceptions of telemedicine (same as page 3)

1. We are interested in understanding breast surgeons’ perspectives on the impact of telemedicine on breast surgical practice, including both positive and negative effects.

Please rate the *effect of telemedicine* on the following factors on a scale of 0-10 (0= very negative effect, 5= neutral, 10= very positive effect)

|  | **Very negative effect** |  |  |  |  | **Neutral** |  |  |  |  | **Very positive effect** |
| --- | --- | --- | --- | --- | --- | --- | --- | --- | --- | --- | --- |
|  | **0** | **1** | **2** | **3** | **4** | **5** | **6** | **7** | **8** | **9** | **10** |
| Use of clinic space |  |  |  |  |  |  |  |  |  |  |  |
| Number of patients seen on a clinic template |  |  |  |  |  |  |  |  |  |  |  |
| Number/length of visits needed per patient |  |  |  |  |  |  |  |  |  |  |  |
| Ability for patients to participate in clinic visits |  |  |  |  |  |  |  |  |  |  |  |
| Establishment/continuation of patient-provider rapport |  |  |  |  |  |  |  |  |  |  |  |
| Capacity to involve patient’s support people in the visit |  |  |  |  |  |  |  |  |  |  |  |
| Ability to integrate multiple staff into visit (eg. medical assistant, nurse, interpreters) |  |  |  |  |  |  |  |  |  |  |  |
| Ability to do multidisciplinary collaboration |  |  |  |  |  |  |  |  |  |  |  |
| Visit documentation |  |  |  |  |  |  |  |  |  |  |  |
| Other (free text) |  |  |  |  |  |  |  |  |  |  |  |
